# Genetic Diversity of MSP1 Block 2 of *Plasmodium vivax* Isolates from Manaus (Central Brazilian Amazon)

**DOI:** 10.1155/2014/671050

**Published:** 2014-02-27

**Authors:** Leidiane Amorim Soares, Janaína Evangelista, Patricia Puccinelli Orlandi, Maria Edilene Almeida, Luciana Pereira de Sousa, Yury Chaves, Roberto Barbosa-Filho, Marcus Vinícius Lacerda, Luis André Mariuba, Paulo Afonso Nogueira

**Affiliations:** ^1^Instituto Leonidas e Maria Deane, Fundação Oswaldo Cruz, Rua Teresina 476 Adrianópolis, 69057-070 Manaus, AM, Brazil; ^2^Universidade Federal do Amazonas, Programa Multi-Institucional de Pós-Graduação em Biotecnologia (PPGBIOTEC) Avenida Rodrigo Otávio Jordão Ramos 3000, Coroado, Manaus, AM, Brazil; ^3^Hospital Fundação de Medicina Tropical Dr. Heitor Vieira Dourado, Avenida Pedro Teixeira 25, Dom Pedro, 69.040-000 Manaus, AM, Brazil

## Abstract

The diversity of MSP1 in both *Plasmodium falciparum* and *P. vivax* is presumed be associated to parasite immune evasion. In this study, we assessed genetic diversity of the most variable domain of vaccine candidate N-terminal PvMSP1 (Block 2) in field isolates of Manaus. Forty-seven blood samples the polymorphism of PvMSP1 Block 2 generates four fragment sizes. In twenty-eight of them, sequencing indicated seven haplotypes of PvMSP1 Block 2 circulating among field isolates. Evidence of striking exchanges was observed with two stretches flanking the repeat region and two predicted recombination sites were described. Single nucleotide polymorphisms determined with concurrent infections per patient indicated that nonsynonymous substitutions occurred preferentially in the repeat-rich regions which also were predicted as B-cell epitopes. The comprehensive understanding of the genetic diversity of the promising Block 2 associated with clinical immunity and a reduced risk of infection by *Plasmodium vivax* would be important for the rationale of malaria vaccine designs.

## 1. Introduction

*P. vivax* remains more widely distributed than *P. falciparum* and is a potential cause of morbidity and mortality amongst the 2.85 billion people living at risk of infection [1]. *P. vivax* malaria accounts for 70% of reported cases in Americas [2]. In Brazil, 202.767 cases of *P. vivax* infection were registered in 2012, corresponding to 85.4% of total cases [3].

At this way, it is extremely important to develop new methods and intervention strategies to block its transmission. One of these alternatives is vaccination, but extensive genetic diversity in natural parasite populations is a major obstacle for the development of an effective vaccine against the human malaria parasite, since antigenic diversity limits the efficacy of acquired protective immunity to malaria [4].

Among the major vaccine candidate antigens, the merozoite protein 1 (MSP1) has been highlighted in several studies which demonstrated their immunogenic potencial [5–13]. In studies conducted in a river side communities, Portuchuelo (Rondonia State), Rio Pardo (Amazonas state), and Ramal do Granada (Acre) from Brazil, using recombinant proteins of Pv-MSP1, it was identified that preferentially the asymptomatic patients had high antibody titers against N-terminal portion of Pv-MSP1, suggesting that protection to this infection may be associated with the presence of these antibodies. Moreover, the acquisition of the repertoire of antibodies against highly polymorphic antigens occurs in individuals exposed to parasite and the clinical protection is induced only after repeated infections [8, 14–16].

The MSP1 gene consists of seven interallele conserved blocks flanked by six variable blocks. Variable blocks show extensive sequence variations consisting of a number of substitutions, insertions, deletions, and varying numbers of short tandem repeats. Between these polymorphic region is the Block 2 repetitive region, from 100 to 400 base pairs (bp) [17].

Merozoite surface protein 1 is the most commonly used genetic marker for the determination of the genetic diversity of the malaria parasite. In some variable blocks, the variation is dimorphic; nonetheless, Block 2 represents an exception to dimorphism and has been used in genetic diversity studies of *P. falciparum* MSP1 [18–23]. Still, *P. falciparum* MSP1 Block 2 has been considered as a potential candidate target for vaccine design [6, 13, 24].

Despite the high potential of the protein, there are no similar studies with ortholog of *P*.* vivax*, the exception is the study performed in western Brazilian Amazon [16]. In order to evaluate genetic diversity of *P. vivax* MSP1 Block-2, PCR amplification was performed with 47 field isolates of *P. vivax* collected in 2009. DNA sequencing analysis was carried out with the positive PCR products. Alleles identified by DNA sequencing were aligned and polymorphism analysis was done by using ClustalW tool in the MEGALIGN program (DNASTAR/Lasergene). We still assess multiplicity of infection to examine distribution of synonymous and nonsynonymous nucleotide substitutions in predicted T and B epitopes. The purpose of this study was to explore the extent of genetic variation in MSP1 Block 2 in central Brazilian Amazon for studying as a molecular marker in epidemiologic investigations and to help in vaccine design.

## 2. Material and Methods

### 2.1. Blood Samples Collection

**Figure 1:**
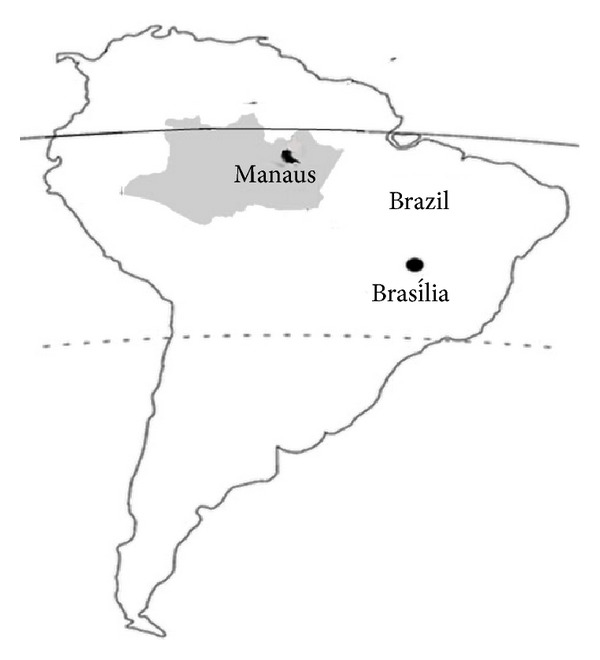
Map showing Manaus, in Central Brazilian Amazon.

Blood samples were collected in 2009 from forty-seven febrile patients diagnosed with malaria for *P. vivax* infection and treated at the Tropical Medicine Foundation of Amazonas a tertiary care centre in Manaus (Figure 1). The study received ethical approval from the Institutional Review Board of the Federal University of Amazonas (Ethical Approval Number 3640.0.000.115-07).

### 2.2. PCR Amplification Products

Genomic DNA was purified by the Charge Switch gDNA 50–100 *μ*L Blood kit (Invitrogen), according to the manufacturer's instructions. We used one pair of oligonucleotide designed by Bastos and colleagues [16] that amplified the longest stretch of variable sequence contained in ICB2-5, Block 2. The primer sense 5′-CTCTGACAAAGAGCTGGAC-3′ was designed based on sequence Block 2 of isolate Belem and annealed to nucleotides 517° to 534°, and antisense 5′-GCTCCTTCAGCACTTTCACGCG-3′ annealed to nucleotides 968° to 989°.

The amplification reactions for Block 2 were performed in a total reaction volume of 50 *μ*L, supplemented with 1 pM primers, 100 *μ*M dNTPs, 1.5 mM MgCl_2_, 1 U of Taq polymerase, and 100 ng of DNA template. The cycling was as follows: one cycle of 95°C for 5 min, followed by 36 cycles of 94°C for 1 min, 63°C for 1 min, 72°C for 1 min, and a last cycle of 72°C for 10 min. The amplicons were visualised in a 1% agarose gel stained with ethidium bromide. The band sizes were determined calculating the ratio of the distance of known bands of 100 bp molecular weight ladder. The PCR products were purified by QIAquick Gel Extraction (Qiagen) according to instructions and frozen at −80°C until shipping for sequencing.

### 2.3. Sequencing

The amplicons of all isolates were shipped on dry ice for sequencing in the facilities of the Program for Technological Development in Tools for Health-PDTIS-FIOCRUZ, located in Salvador, BA, Brazil. The sequencing reactions were performed in automatic DNA MegaBace 1000 by the dideoxy method. Only the amplicons with optimal concentration were sequenced. The high-quality sequences were chosen by the Phred program. The electropherograms were visualised and edited in EditSeq of Lasergene packet, version 4.05 (DNAStar). Nucleotide and amino acid sequences were compared with the corresponding accessible sequences of GenBank by Blast-P from the National Centre for Biotechnology Information to select those PvMSP1 Block 2 sequences which had higher similarities, more than 90%. From each isolate, the consensus PvMSP1 Block 2 sequences were edited, comparing duplicate sequencing of both sense and antisense strands. They were submitted to GenBank search to select two or three other PvMSP1 sequences worldwide with the most similarity and one or two sequences with less similarity, according to Blast-P estimation. The nucleotide sequences were deposited in GenBank under submission number HQ200196-HQ200223.

### 2.4. Mixed Clonal Infections by Cloning of PCR Products

The purified fragments were ligated into the TOPO cloning vector (Invitrogen). The ligations were conducted at a temperature of 16°C overnight (following the manufacture protocol) and then introduced into *Escherichia coli* (Top-10 strain) by thermal shock. Nine colonies were expanded and extracted using a mini prep kit (Qiagen). The purified plasmids were then sequenced using the sense and antisense primers targeting Block-2 PvMSP1. The amplicons were sequenced in an automatic DNA MegaBace 1000 using the dideoxy method.

### 2.5. Gene Analysis

The sequences were analyzed using the PHRED and CAP3 software tools for the correction of possible errors and to provide the electropherograms graphics. For the investigation of multiple clones of *P. vivax* infection, we aligned the various sequences using the EditSeq program and the MegAlign Lasergene package, version 4.05 (DNA Star). The editing of the sequences, conceptual translations, and amino acid alignments were performed using the EditSeq and MegAlign programs of the DNAStar package (Lasergene). A multiple alignment was performed with two isolates (10 and 15) using MUSCLE and gaps were considered as lost data. Using Blast to check for similarity among them the haplotypes of isolate 10 were most similar to GQ890943 sequence from Thailand and haplotypes of isolate 15 with AF435623 from Brazilian Amazon.

### 2.6. Prediction of Linear B- and T-Cell Epitopes

B-cell epitope predictions were carried out on 24 amino acids of PvMSP1 Block 2 using the BepiPred 1.0 Server [25]. Putative epitopes that were 12 amino acids in length were generated with a specificity of 75%. Subsequently, differential binding of T-cell epitopes spanning the Block 2 fragments was predicted using the ProPred MHC class II binding peptide prediction server [26] for four MHC class II HLA alleles, including HLA—DRB1*0101, DRB1*0401, DRB1*0701, and DRB1*1101.

## 3. Results

### 3.1. Polymorphism of PCR Products of PvMSP1 Block 2

**Figure 2:**
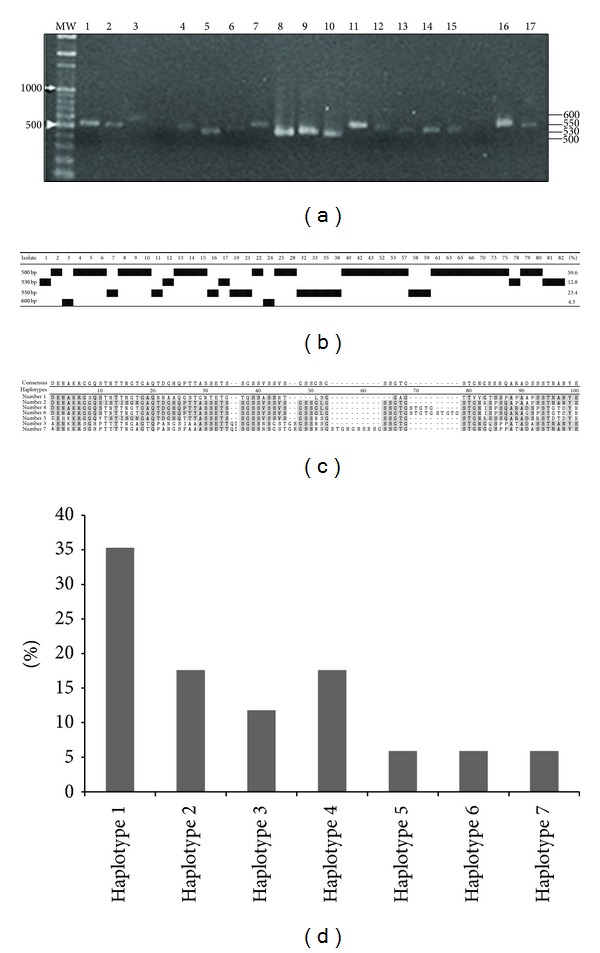
Analysis of diversity of PCR products of *PvMSP1* Block 2. (a) Agarose gel showing fragment size of PCR products of the PvMSP1 Block 2 in 17 samples. Based on 100 bp molecular weight ladder (MW), four different types of fragments ranging, and 500, 530, 550 between 600 base pairs were defined by calculating the ratio of the distance of known bands (right side). (b) Distributions of fragments per isolate and frequencies of each type of fragment are shown. (c) Based on amino acid sequences alignment of *PvMSP1* Block 2, seven haplotypes could be classified by short tandem in positions 10° to 70°. At the top of the alignment is consensus sequence. (d) Prevalence of seven haplotypes among field isolates in Manaus.

Of forty-seven blood samples, the polymorphism of PvMSP1 Block 2 generated one fragment size which ranged between 500, 530, 550, and 600 base pairs, as seen by agarose gel (Figure 2(a)). The 500 bp fragment was the most frequent among isolates (Figure 2(b)). Sequencing of Block 2 was performed with twenty-eight *P. vivax* of these isolates and deposited in GenBank under submission number HQ200196-HQ200223. Seven haplotypes could be classified by short tandem in positions 10° to 70° amino acids (Figure 2(c)) and their prevalence was determined (Figure 2(d)).

Only an apparent tandem degenerating 5-mer repeat (GSXXX) has been described in the Block 2 [17]. One more detailed analysis showed that this degenerating 5-mer repeat may be expanded into two types of short tandem repeats. The first presented in the form of a degenerating tripeptide repeat SSX (where X stands for E, G, T, A, N, P, V, or S residue) and a conserved tripeptide repeat GST (Table 1). Some of these repeats were synonymous substitutions as is the case of SSG (four combinations of degenerate codons), SSV, and SSN (both with two combinations) and lastly the codon degeneracy of GST had three types. Some of them ranged in numbers of repetitions (Table 1). These short tandem repeats were present in all haplotypes and facilitated the distinction of them.

The haplotypes identified in Manaus were similar to amine acid sequences from other regions (Table 2). The most predominant sequence, the haplotype number 1 (Figure 2(d)), was very similar to *Belem* one and other southeastern Asian sequences from Bangladesh, Vanuatu, and Sri Lankan. The mild sequences, haplotypes number 2, number 3, and number 4, were also detected in South Korea, Bangladesh, Thailand, and Brazil. The minor sequences number 5, number 6, and number 7 were also detected in the same localities. Still, the sequence PvMSP1 Block 2 of haplotype number 7 was similar to that of strain *Sal-1*. The existence of the same MSP1 Block 2 haplotypes should be important for the rationale of malaria vaccine designs.

### 3.2. Intra-Allele Recombination in PvMSP1 Block 2

**Figure 3:**
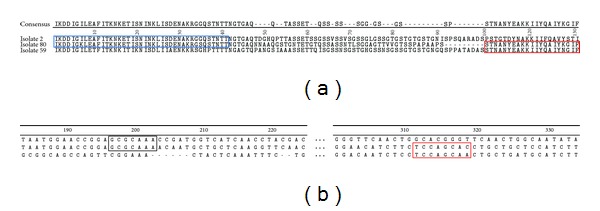
Evidence of intra-allele recombination in PvMSP1 Block 2. (a) Alignment of sequences of isolates (2, 80, and 59) showing exchange of homologues sequences. The segment of isolate 80 could have been originated from others isolates; blue rectangle shows exchange between isolates 2 and 80, red rectangle exchange between isolates 59 and 80 and (b) DNA-SP 5.0 analysis determined two putative recombination sites (dark and red boxes) in the *PvMSPI* gene for generating the isolate 80.

Of twenty-eight amino acid sequences, evidence of striking exchanges was observed with two stretches flanking the repeat region of isolate 80 (Figure 3(a)). The upstream sequence IKDDIG-LEAFITKNKETTISNINKLSDENAKRG-QSTNT was similar to isolate 2. In the same isolate, another recombination event was observed in the downstream sequence SSTNANYEAKKIIYQAIY-GIFTNQLEEA similar to isolate 59.

Two predicted recombination sites, GCGCAAA (or its complementary sequence CGCGTTT) and TCCAGCAC (or its complementary reverse sequence GGTCGTGG), were observed (Figure 3(b)). The last predict recombination site is very similar to the Chi sequence (**G** C **T** G **GTGG**), which locally increases recombination in *Escherichia coli* and is merged of CAGGTG, a predicted recombination site from hypothetical progenitors and RO33 and MAD20 haplotypes of *P. falciparum* [29]. These data demonstrate a predicted recombination site in *P. vivax* MSP1.

### 3.3. Single Nucleotide Polymorphisms in PvMSP1 Block 2

**Figure 4:**
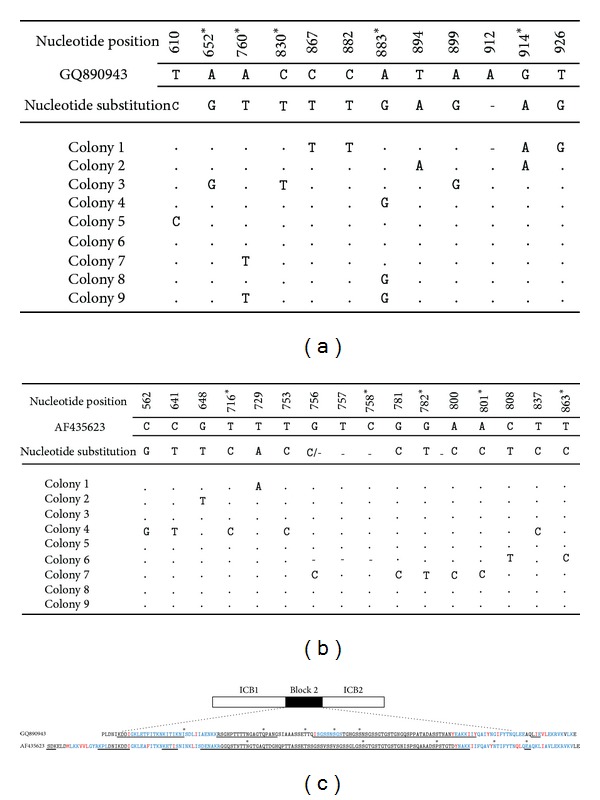
Occurrence of nucleotide diversity by non synonymous and synonymous mutations in polymorphic region of Block 2. (a) Using Blast, the sequence GQ890943 was similar to the haplotypes of isolate 10. Only nucleotide substitutions were shown in the panel with colonies of *E. coli* containing PCR products of Block 2 from isolate 10 cloned into plasmids. (b) The same was evaluated with PCR products of Block 2 from isolate 15. The sequence AF435623 was selected using BLAST and only nucleotide substitutions were shown in the panel. (c) The sequences encompassing Block 2, (interspecies conserved blocks (ICB) 1 and 2) were represented. Prediction of linear B-cell epitopes was carried out and underlined by bars [25]. Differential binding of T-cell epitopes was predicted for all HLA-DRB alleles accessible into the ProPred using MHC class II binding peptide prediction server [26]. We observed several T-cell epitopes (blue letters) with residue anchor (red letter). Location of nonsynonymous substitutions was represented by black dots. Superior sequence (GQ890943) was similar to haplotypes of isolate 10. Inferior sequence (AF435623) was similar to haplotypes of isolate 15.

In order to evaluate the occurrence of multiclonal infections, sequencing of PCR products cloned into plasmid was performed in the isolates 10 and 15 (Figure 4). The sequence GQ890943 was similar to the haplotypes of isolate 10 and served as template. Only nucleotide substitutions were shown in the panel with colonies of *E. coli* containing PCR products of Block 2 from isolate 10 cloned into plasmids (Figure 4(a)). Eight haplotypes were identified from nine colonies of isolate 10 that presented at least a single nucleotide mutation. In total, eleven dimorphic nucleotide substitutions were observed of which five were by nonsynonymous substitutions (asterisk in Figure 4(a)).

The same was evaluated with PCR products of Block 2 from isolate 15. The sequence AF435623 was selected using BLAST and only nucleotide substitutions were shown in the panel (Figure 4(b)). Five haplotypes were identified in the isolate 15 after determination of sixteen single nucleotide polymorphisms (SNPs), whereas five of them were by nonsynonymous substitutions (asterisk in Figure 4(b)).

Punctual substitutions in polymorphic Block 2 of the *MSP1* gene were random and frequent events but preferentially distributed in the repeat rich region. We observed that nonsynonymous substitutions were more frequent in the B-cell epitopes (underlined letters) than in T-cell epitopes with residue anchors (blue and red letters) within of Block 2 (Figure 4(c)). The occurrence of nonsynonymous substitutions supports the idea that Block 2 is continually evolving under immune selective pressure.

## 4. Discussion

Different studies suggest that acquisition of antibodies against the domain Block 2 of ortholog MSP1 could associate with clinical immunity and a reduced risk of infection with *Plasmodium vivax* [8, 14, 15]. Nonetheless, highly specific antibodies against to allelic types of MSP1 Block 2 are non-cross-reactive, and notably, this extensive allelic diversity of MSP1 may impede the development of effective vaccines. And hence, antibodies against multiple MSP1 Block 2 alleles would be needed to protect against the maximum number of parasites, taking into account the divergent sequences that occur naturally [30].

However the alignment of several PvMSP1 alleles of the Block 2 possessed an apparent tandem degenerating 5-mer repeat (GSXXX) that could range from 0 to 9 repetitions [17]. Here, we present information about genetic diversity of MSP1 Block 2 of isolates of* Plasmodium vivax* circulating in Manaus (Brazilian Amazon). Initially, four sizes of PCR products ranged from 500 to 600 base pairs were amplified from blood samples (Figure 2(b)). Nonetheless, after sequencing of Block 2 of twenty-eight *P. vivax* isolates, extensive sequence variations consisting of a number of substitutions, insertion, and deletions and varying numbers of short tandem repeats were found (Figure 2(c) and Table 1) such that seven distinct variants were identified circulating in endemic area from Manaus. These results confirm what had been suggested with MSP1 Block 2 of *P. falciparum* at which the fragment size may not be an accurate marker for genetic diversity within MSP1 Block 2 [22].

Regarding the PvMSP1 Block 2 sequences found in Manaus, two of them were similar to major *Belem* and *Sal-1* haplotypes, number 1 and number 7, respectively. According to our data, *Belem* haplotype is predominant among *P. vivax* isolates circulating in Manaus. All haplotypes identified were common to other malaria-endemic areas (Table 2), South Korea, Thailand, Bangladesh, Vanuatu, Sri Lankan, and Brazil [16, 17, 27, 28, 31]. As the Block 2 region of PvMSP1 has been considered a promising new candidate for the development of a malaria vaccine, as it is a target of protective immunity against P. vivax [14, 15], the existence of same MSP1 Block 2 haplotypes in different malaria endemic areas will be important for the rationale of malaria vaccine designs.

This study also demonstrated evidence of recombination in polymorphic Block 2 in three isolates, describing predicted recombination sites in genome of *P. vivax*. One of them is very similar to the Chi sequence, which locally increases recombination in *Escherichia coli* [32]. Another predicted recombination site would be merging to hypothetical progenitors and RO33 and MAD20 haplotypes of *P. falciparum* [29, 33]. Intragenic recombination during meiosis has been proposed as an important mechanism for the generation of new genetic variants on malaria antigens, and it was also one of the most important factors considered to explain the generation of new alleles in the MSP1 context [22, 34].

Studies evaluating number of concurrent infections per patient or MOI (multi clonal infection) have been used as one of several measures of the impact of malaria intervention [35]. Importantly, sequence analysis revealed Block 2 as a hot spot for genetic variation. The non-synonymous substitutions were preferentially distributed in the rich-repeat region that contained the B-cell epitopes predicted by BepiPred [25]. Based on genetic diversity in *Plasmodium falciparum* merozoite surface proteins, nonsynonymous SNPs contribute largely to the variability of the parasite and provide escape from host immunity [34, 36].

In conclusion, the generation of diversity of the most polymorphic block from orthologs MSP1 accumulates recombination sites and multiples nonsynonymous substitutions. Based on findings acquisition of antibodies against the MSP1 Block 2 could associate with clinical immunity and reduced risk of infection with *Plasmodium vivax*; a comprehensive understanding of genetic variation of the promise malaria vaccine candidate would be important for the rationale of malaria vaccine designs.

## Figures and Tables

**Table 1:** Repeats and their patterns of codon degeneracy in Pv-MSP1 Block 2 haplotypes.

Tripeptide repeats	Haplotypes							Pattern of codon degeneracy
	1	2	3	4	5	6	7	
SSE	1^1^	1^1^	1^1^	1^1^	1^1^	1^1^	1^1^	1-AGT-TCG-GAA
SSG	0	3^1,2,3^	1^3^	3^1,2,3^	2^1,3 or 4^	3^1,2,3^	1^3^	1-TCT-TCT-GGA
								2-TCA-TCT-GGC
								3-TCG-AGT-GGC
								4-TCC-TCT-GGA
SSV	0	2^1,2^	0	2^1,2^	2^1,2^	2^1^	0	1-AGT-TCT-GTT
								2-TCA-TCT-GTC
SST	1	1	1		1		1	1-TCT-TCA-ACA
SSA	1							1-AGT-TCT-GCT
SSN	1^2^		1^1^		1^1^		1^1^	1-TCG-TCT-AAC
								2-TCA-TCT-AAC
SSP	1							1-TCT-TCT-CCA
SSS					1			1-TCA-TCT-TCT
GST	1^3^	1^1^	2^2^	2^1^	1^1^	3^1^	3^3^	1-GGT-TCA-ACT
								2-GGT-TCG-ACT
								3-GGT-TCA-ACA

Numbers indicate how many repetitions one tripeptide repeat occurred in each haplotype. The overwritten numbers distinguish synonymous mutations in the codon sequences of repeat presented in each haplotype, according to column Pattern of codon degeneracy.

**Table 2:** Similarities between amino acid sequences of Manaus haplotypes and others regions.

Haplotype	Similarity	Accession number	Origin	References
1 accession number: AEA77298	96%	AAN86210	Bangladesh	[17]
	100%	AAN86238	Brazil	[17]
	93%	AAN86243	Vanuatu	[17]
	90%	AAA63427 (Belem)	Para (Brazil)	[27]
	99%	CAA40355	Sri Lankan	[27]

2 accession number: AEA77275	100%	AAN86221	Thailand	[17]
	100%	ADF48579	Thailand	[28]

3 accession number: AEA77282	99%	AAN86235	Thailand	[17]
	100%	ABV25925	Acre (Brazil)	[16]
	94%	AAN86229	Bangladesh	[17]

4 accession number: AEA77292	100%	ABV25923	Acre (Brazil)	[16]
	100%	ADF48559	Thailand	[28]
	94%	ADF48816	South Korea	[28]

5 accession number: AEA77272	100%	AAN86231	South Korea	[17]
	100%	AAN86237	Brazil	[17]

6 accession number: AEA77293	99%	ADF48790	Thailand	[28]
	99%	AAN86213	Thailand	[17]

7 accession number: AEA77276	99%	AAM22837	South Korea	Han and Chai, 2001, unpublished
	82%	EDL45115 (sal-1)	Salvador	Carlton J., unpublished
	92%	AAN86232	Bangladesh	[17]
	100%	AAN86246	Thailand	[17]

Haplotypes: accession number in GenBank of amino acid sequences from haplotypes.

Similarities determined by Blast-P program.
